# HIV retesting prevalence among clients accessing anti-retroviral therapy and HIV testing services in Ghana

**DOI:** 10.1371/journal.pone.0316915

**Published:** 2025-02-21

**Authors:** Raphael Adu-Gyamfi, Stephen Ayisi Addo, Nyonuku Akosua Baddoo, Ernest Kenu, Anthony Ashinyo, Kwadwo Koduah Owusu, Menard Laurent Chihana, Alice Sallar Adams, Odikro Magdalene, Delia Bandoh, Marijanatu Abdulai, Kenneth Danso, Kafui Senya, Cheryl Johnson

**Affiliations:** 1 National AIDS/STI Control Programme, Ghana Health Service, Accra, Ghana; 2 Department of Community Health, University of Ghana Medical School, Accra, Ghana; 3 Department of Epidemiology and Disease Control, University of Ghana School of Public Health, Accra, Ghana; 4 World Health Organization Global HIV, Hepatitis and STI Programmes, Geneva, Switzerland; 5 World Health Organization, Communicable and Non-Communicable Disease Cluster, Accra, Ghana; University of Zimbabwe Faculty of Medicine: University of Zimbabwe College of Health Sciences, ZIMBABWE

## Abstract

**Introduction:**

Ghana is working towards achieving the 95-95-95 targets for its HIV response. One challenge has been low linkage to care rates, possibly due to high rates of retesting among people living with HIV who are already aware of their status. This leads to an overestimation of the first 95 and a subsequent underestimation of the second 95. This study aimed to measure the prevalence of HIV retesting among PLHIV in Ghana who are already aware of their status and to explore their reasons for retesting.

**Methods:**

This was a facility-based cross-sectional study conducted in the three ecological zones of Ghana. A total of 11,145 individuals from 30 ART centres and 90 HTS centres participated. The sample size for each zone was determined proportionally based on the number of people enrolled in ART. A profiling tool was used to assess testing behaviours among clients visiting HTS sites linked to ART clinics. Focus group discussions were also conducted with clients and health workers to gather their perceptions of reasons for retesting.

**Results:**

Participants were predominantly female (74.3%; 8,285/11,145), with a median age (interquartile range) of 43.0 (35–52). The prevalence of retesting among ART clients was 32.9% [95% CI: 0.32–0.34] (3,670/11,145). Among those who retested, the majority did so twice (2,041; 55.6%). Of the clients who tested positive for HIV during the study period, 53.1% (43/835; 95% CI: 0.49–0.57) had a previous HIV diagnosis. Adjusting for retesting, the positivity rate at HTS sites decreased from 8.4% to 4.1%. Key reasons for retesting included the desire to confirm diagnosis, denial and doubt regarding test results, retesting required due to documentation issues, and religious beliefs.

**Conclusion:**

The prevalence of retesting over the past six years was found to be high, resulting in an overestimation of HIV positivity rates and affecting linkage to care. Implementing interventions to accurately account for retesting instances may improve data accuracy and the country’s linkage to care rate, bringing Ghana closer to achieving the 95-95-95 targets.

## Introduction

Achieving the UNAIDS 95-95-95 targets is crucial for achieving and maintaining a low HIV incidence by 2030. In 2017, Ghana developed a national strategy to accelerate progress towards these global targets. However, national program reviews in 2019, revealed gaps in knowledge of HIV status among people living with HIV (PLHIV) (43%) [1] and low linkage to care rates defined as proportion of people living with HIV initiated on ART (55%) [2]. As a result, differentiated services for testing and treatment were implemented. During the implementation of these new programs, it became apparent that several HIV testing and treatment sites were reporting an increasing number of retests among individuals who had previously been diagnosed with HIV.

Retesting in the context of this study, refers to the situation where an individual who already knows their HIV positive status, goes back to an HIV testing facility to get another HIV test without necessarily disclosing they already know their positive status.

Similar trends have been reported in multiple countries as antiretroviral therapy coverage increases [3–5]. These countries have faced challenges and concerns regarding how best to support retesting patients as well as how to address retesting given their limited resources for service delivery. Various studies conducted in sub-Saharan Africa have reported a prevalence of retesting among PLHIV ranging from 2% to 68% [4, 6–9]. For example, a study in Ethiopia found that 13.2% of 831 ART clients reported retesting at least two weeks prior to starting care [7]. In Tanzania, 2.0% of all men at a voluntary counseling and testing site indicated that they had previously been diagnosed with HIV [4], while other studies reported much higher prevalence rates of HIV retesting [6, 8, 9]. The reasons provided by clients who undergo retesting include the need to confirm the diagnosis for those who initially doubt their diagnosis and having no symptoms prior to diagnosis [4, 7, 10–12]. Among those already enrolled on ART, some individuals undergo retesting for reasons such as feeling better after starting ART [11] or believing they have been cured by spiritual powers [6] or other reasons.

Understanding retesting is important to ensure that patient needs are met during service delivery and to adapt programs to support reengagement in care when necessary. Moreover, without an understanding of the extent of retesting among people with HIV, it is difficult to gauge progress towards the first and second UNAIDS 95 95 95 global targets. The challenge is especially so for countries such as Ghana with limited unique identification systems for HIV testing services. These gaps also hinder the ability of many national programs to effectively target efforts to optimize HIV services.

Therefore, in this study, we report the results of a mixed methods approach, including a survey and focus group discussions. Our objectives were to determine the prevalence of retesting among people living with HIV (PLHIV) and to gain insights for optimizing programming for retesters in Ghana. This knowledge will enhance the availability of data on linkage to care, which can be used to formulate appropriate policies. An understanding of the prevalence of retesting especially amongst persons already on ART is useful for improving the accuracy of linkage to care rates.

## Methods

### Study design

This study was a facility-based cross-sectional study employing both quantitative and qualitative methods of data collection.

### Study setting and population

Ghana is a West African country which lies between latitude 5 and 11 degrees north and occupies an area of 239 460 sq. km. It is bordered by Côte d’Ivoire on the west, Burkina Faso in the north, Togo on the east, and the Atlantic Ocean in the south. The country’s 2020 population was projected at 31 072 940 [13]. Ghana is divided into three ecological zones: the Guinea savanna zone (the northern belt), the transitional zone (the middle belt) and the forest zone (the southern belt). The country is further divided into 10 administrative regions namely Northern, Upper east, Upper west, Ashanti, Brong Ahafo, Western, greater Accra, Volta, Central, and Eastern region.

Ghana has a generalized HIV epidemic with its 2020 national HIV prevalence among adults 15–49 years old estimated at 1.7%, higher among women than men [14]. The study was implemented in two different settings, 1. ART centres and 2. HTS centres. For recruitment at ART centres, people were eligible to participate in the study if they were adults 18+ years living with HIV who were already on ART and accessing treatment services at one of the selected facilities. For recruitment at HTS centres, eligibility was all 18+ years old adult individuals seeking HTS at the selected HIV testing centres across country. HIV testing services are provided in almost all 5000 health care facilities across the country, while treatment services were provided in about 600 ART clinics. Participants were PLHIV accessing either HIV testing or treatment at facilities across three sub-national zones within Ghana: northern, middle and southern. Children and persons who did not consent were excluded from the study.

### Sample size and sampling method

The study was conducted in randomly selected thirty ART facilities and ninety HIV testing centers across the three zones of the country. From each zone, 10 health facilities were selected comprising of one teaching hospital, two regional hospitals, three district hospitals and four health centres. Each selected facility contributed one study site offering ART services per facility per zone. Regarding number of HIV testing centres, each teaching hospital contributed 10 HTS centres, the 2 regional hospitals contributed a combined total of 10 HTS centres together, the 3 district hospitals contributed a combined total of 5 HTS centres together and the 4 health centres contributed a combined total of 5 HTS centres per zone (Table 1). A total sample size of 11,000 was calculated, covering 10% of the 110,000 clients enrolled on ART as at March 2019 in Ghana. The sample size for each zone was calculated, proportionate to ART case load. Teaching hospitals were allotted 40%, regional hospitals 20%, district hospitals 5% and health centers 1.25%. PLHIV who access ART services in the study facilities were consecutively interviewed until the sample size for each facility was attained.

**Table 1:** Types of facilities selected from each zone.

	Type of Service Offered	
Type of Facility	ART	HTS
Teaching hospital	1	10
Regional hospital	2	10
District hospital	3	5
Health center	4	5
**Total**	**10**	**30**

### Data collection methods and tools

Data were collected from 21st October 2019 to 19th December 2019 during clinic days, which is no more than three clinic days per week. At sampled facilities, trained data collectors would approach a client visiting the ART clinic to collect their antiretrovirals (ARV), explain the study to them and seek their consent to be enrolled into the study. If they were willing and consented to participate in the study, a semi-structured questionnaire was administered to them. The questionnaire contained among other questions on clients’ demographics, testing attributes and whether the client is currently enrolled on ART if already positive. Similarly, among clients who visited the HTS centres, information about the study was explained to them. For those who were interested to participate in the study and had given consent, a profiling tool was administered to them by trained counsellors. The profiling tool had a series of questions that were asked to clients testing for HIV to determine their testing behaviours and history of testing. The profiling tool was administered to identify anyone who was retesting. It was conducted as part of the routine HIV testing and counselling session by the counsellors. Two focus group discussions (FGDs), one for clients and the other for health workers, were also conducted in each zone, to provide more insight into reasons for HIV retesting.

### Data processing and analysis

All Data from ART centres and profiling questionnaires were entered into ODK. Data was downloaded in Microsoft Excel format and cleaned. Cleaning focused on missing and out of range variables. An Excel sheet of issues arising in the data was shared and corrections were entered manually. Cleaned data was exported to STATA version 15 for analysis. Data analysis focused on proportions. Proportions of demographics were generated and prevalence of retesting determined.

Retesting prevalence was defined as below:

$$\frac{No\;of\;confirmed\;HIV\;positive\;persons\;who\;reported\;retesting\;with\;same\;results*100}{Total\;number\;of\;PLHIV\;interviewed}$$

The prevalence was further disaggregated by other relevant characteristics of respondents including demographics such as sex and age, year, and location of interview (ART vs HTS). Clients who could not provide their exact year of diagnosis were excluded from the calculation of the annual retesting prevalence (except for 2019).

All results are presented using tables and figures. FGDs were transcribed verbatim and analysed using content analysis. A rational analysis was employed as we had predetermined themes, primarily to understand the reasons behind why people retest, and to connect these findings with the results of the quantitative data analysis. After the data was transcribed, the approach became more flexible; we selected quotes that aligned with the predetermined themes in the transcribed data. These quotes are presented under the defined themes.

### Ethical considerations

Ethical clearance was obtained from the Ethical Review Committee of the Ghana Health Service (GHS-ERC 023/08/19) before commencement of the study. Permission was also sought from the National AIDS/STI Control Program (NACP), regional and district health directorates and the management of the various facilities. Study participation was strictly voluntary. Participants were educated on the purpose of the study and its objectives and asked to sign written informed consent forms before being enrolled into the study. All questions/concerns were duly addressed.

## Results

A total of 11,145 PLHIV accessing ART (30 facilities) and 9,975 accessing HIV testing services (90 facilities) were interviewed across the three zones of Ghana. Out of the 9,975 visiting the HTS centers, 835 were diagnosed positive and were therefore profiled. The highest number of interviews were in the southern zone (5,305 (47.6%)), while the middle zone had the highest number of profiles done (Table 2).

**Table 2:** Distribution of interviews and profiles conducted at the ART and HTS centers, HIV retesting survey, Ghana, 2019. n = 11,145.

Characteristic	Clients at ART sites (n = 11,145)	Clients at HTS sites (n = 835)
	n (%)	n (%)
Zone		
Northern	991 (8.9)	166 (19.9)
Middle	4,849 (43.5)	394 (47.2)
Southern	5,305 (47.6)	275 (32.9)
Region		
Northern	651 (5.8)	135 (16.2)
Upper East	272 (2.4)	22 (2.6)
Upper West	68 (0.6)	9 (1.1)
Ashanti	2,354 (21.2)	207 (24.8)
Brong Ahafo	1,509 (13.5)	115 (13.7)
Western	986 (8.9)	72 (8.6)
Greater Accra	2,273 (20.4)	129 (15.5)
Volta	1,445 (12.9)	37 (4.4)
Central	163 (1.5)	2 (0.3)
Eastern	1,424 (12.8)	107 (12.8)
Type of Facility		
Tertiary	4,769 (42.8)	366 (43.8)
Regional	4,841 (43.4)	263 (31.5)
District	1,246 (11.2)	144 (17.3)
Health Center	289 (2.6)	62 (7.4)

### Demographic characteristics of clients at ART sites enrolled on ART

Distribution of demographic characteristics of study participants are presented in Table 3. The majority of clients interviewed at the ART centers were female—74.3% (8,285/11,145), most between 41–50 years -32.3% (3,598/11,145). The median age at ART sites was 44 (36–52) with the ages ranging from 18 to 79 years. Most clients interviewed were diagnosed in 2011 to 2019–61.9% (6,902/11,145). At the HTS centers, females were also in the majority (66.1%) and the median age group was 36 (29–46).

**Table 3:** Demographic characteristics of clients interviewed and profiled, HIV retesting survey, Ghana, 2019, n = 11,145.

Characteristic	Clients at ART sites (n = 11,145)	Clients at HTS sites (n = 835)
	n (%)	n (%)
Sex		
Male	2,860 (25.7)	283 (33.9)
Female	8, 285 (74.3)	552 (66.1)
Age		
18–30	1,364 (12.2)	250 (29.9)
31–40	3,010 (27.0)	267 (32.0)
41–50	3,598 (32.3)	186 (22.3)
51–60	2,265 (20.3)	95 (11.4)
>60	900 (8.1)	28 (3.4)
Don’t Know	8 (0.1)	7 (1.0)
Year of Diagnosis		
1995–2000	44 (0.4)	
2001–2010	2,968 (26.6)	
2011–2019	6,902 (61.9)	
Unknown	1,231 (11.1)	

### Retesting prevalence

Overall, the prevalence of retesting at the HTS centers was found to be higher 53.1% (95%CI: 0.50–0.56) than at the ART clinics, which was at 32.9% (95% C.I: 0.32–0.34). Distributions of retesting prevalence by zone, region, age and sex are shown in Table 4.

**Table 4:** Distribution of retesting prevalence at ART and HTS centers, HIV retesting survey, Ghana 2019.

Characteristic	Clients at ART sites			Clients at HTS sites		
	No. of Retesters	No. of Respondents	Prevalence	No. of Retesters	No. of Respondents	Prevalence
**Retesting prevalence**						
**Ever retested**	3,670	11,145	32.9	443	835	53.1
**Never retested**	7,475	11,145	67.1	392	835	46.9
**Zonal ever retested prevalence**						
**Southern**	1,709	5,305	32.2	180	275	65.5
**Middle**	1,603	4,849	33.1	164	394	41.6
**Northern**	358	991	36.1	99	166	59.6
**Regional ever retested prevalence**						
**Ashanti**	611	2,354	26.0	66	207	31.9
**Brong-Ahafo**	542	1,509	35.9	57	115	49.6
**Central**	66	163	40.5	2	2	100
**Eastern**	425	1,424	29.8	85	107	79.4
**Greater Accra**	977	2,273	43.0	77	129	59.7
**Northern**	259	651	39.8	74	135	54.8
**Volta**	241	1,445	16.7	16	37	43.2
**Upper East**	87	272	32.0	22	22	100
**Upper West**	12	68	17.6	3	9	33.3
**Western**	450	986	45.6	41	72	56.9
**Retesting prevalence by sex**						
**Male**	973	2,860	34.0	150	283	53.0
**Female**	2,697	8285	32.6	293	552	53.1
**Ever retested prevalence, by age group**						
**18–30**	533	1,364	39.1	533	1,364	39.1
**31–40**	1,087	3,010	36.1	1,087	3,010	36.1
**41–50**	1,128	3,598	31.4	1,128	3,598	31.4
**51–60**	703	2,265	31.0	703	2,265	31.0
**>60**	219	908	24.0	219	908	24.0

**Fig 1:**
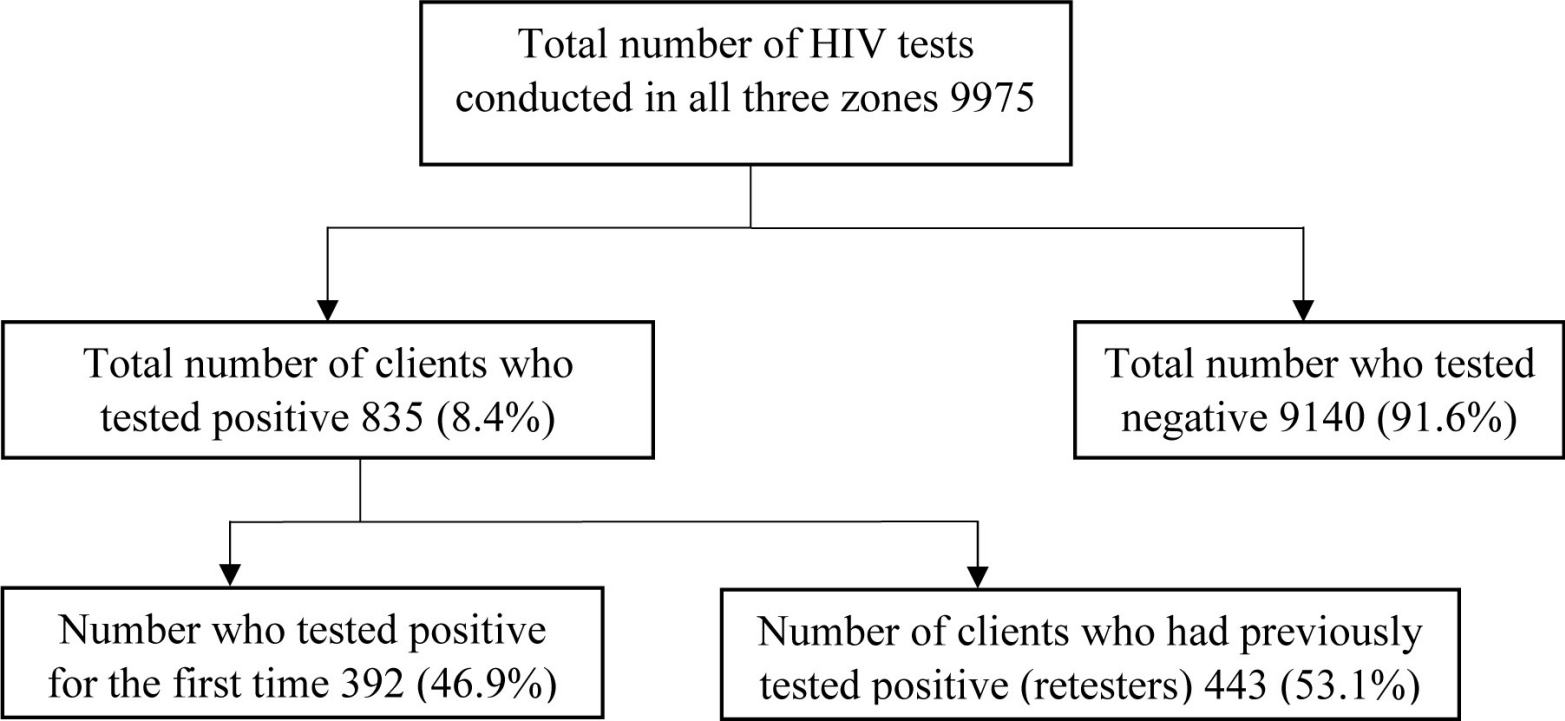
Flow diagram showing HIV tests conducted and retesting at HTS sites, Ghana, 2019.

Out of the 835 clients who tested positive at the HTS sites across the country, 443 reported ever testing for HIV previously. This gave an HTS profiling retesting prevalence of 53.1% and a positivity rate of 8.4% (835/9975), however after adjusting for retesting the actual positivity rate would be 4.1% (392/9532) (Fig 1).

### Number of times retested (2014–2019)

From 2014 to 2019, the highest percentage of retesting at both ART and HTS centers was for those who retested once (Table 5).

**Table 5:** Number of times clients retested, 2014–2019.

**Clients at ART sites**						
**Characteristics**	**Frequency of retesting (%) among those who reported retesting in a particular year**					
Number of Times Retested	**2014 (N = 307)**	**2015 (N = 315)**	**2016** **(N = 396)**	**2017 (N = 505)**	**2018 (N = 714)**	**2019 (N = 985)**
1	173 (56.4)	213 (67.6)	267 (67.4)	310 (61.3)	448 (62.7)	617 (62.6)
2	103 (33.6)	79 (25.1)	94 (23.7)	159 (31.5)	216 (30.3)	299 (30.4)
3	22 (7.2)	17 (5.4)	27 (6.8)	26 (5.1)	40 (5.6)	63 (6.4)
4	6 (1.9)	4 (1.3)	6 (1.5)	7 (1.4)	7 (0.9)	3 (0.3)
5	2 (0.6)	2 (0.6)	2 (0.6)	1 (0.2)	3 (0.4)	2 (0.2)
6	1 (0.3)	-	-	1 (0.2)	-	-
>8	-	-	-	1 (0.2)	-	1 (0.1)
**Clients at HTS sites**						
**Characteristic**	**Frequency of retesting (%) among those who reported retesting in a particular year**					
**Number of Times retested**	**2014 (N = 15)**	**2015 (N = 23)**	**2016 (N = 47)**	**2017 (N = 67)**	**2018 (N = 111)**	2019 (N = 286*)
1	11 (73.3)	16 (69.8)	31 (65.9)	48 (71.6)	73 (65.8)	177 (61.8)
2	2 (13.3)	5 (21.7)	12 (25.5)	15 (22.4)	30 (27.0)	79 (27.6)
3	1 (6.7)	1 (4.4)	2 (4.3)	1 (4.5)	4 (3.6)	23 (8.0)
4	-	-	-	-	2 (1.8)	6 (2.1)
>5	1 (6.7)	1 (4.4)	1 (4.4)	1 (1.5)	2 (1.8)	1 (0.4)

### Annual retesting prevalence (2014–2019)

For the past six years, the annual prevalence of retesting among clients at the ART centres for the year a client was diagnosed positive was as follows: 24.1% (2014), 25.0%, 23.5%, 22.3%, 24.9% and 24.3% (2019). In comparison, the prevalence among all retesters during the same years was 8.1% (2014), 7.4%, 8.2%, 9.1%, 11.3%, and 8.8% (2019).

### Identifiers used by retesters at HTS sites (2014–2019)

The majority of clients who retested (2998/3222 (93%) and 517/549 (94.2%) for ART and HTS centers respectively) used their same names but mainly did not inform service providers they were retesting.

### Reasons for retesting

Of respondents who stated reasons for retesting in the past 12 months, most reported that the reason for retesting was to confirm their results (854 respondents). This was confirmed from both client and health worker discussions.

*The reasons why people do retesting is mostly for confirmation, they want to compare the confirmed first and second results to be sure*” (Client FGD–Northern Zone).

“*What I have realized is that*, *they want to be sure and for confirmation*” (Health worker FGD–Southern Zone).

“*What I realized is that*, *those who test at the smaller facilities tend not to believe the results we tell them till they come to Korle Bu to confirm it*” (Health worker FGD–Southern Zone).

This was followed by retesting due to denial and doubt (307). Respondents reported that clients who test positive initially remain in denial. This causes them to retest.

*I have done retesting before. I did my first test in 2001. I did a second in 2003. Reason being that, I didn’t accept the results of 2001 and in 2003, I was still strong so I chose to be retested. After I was again tested positive, I finally came to accept it*”–(Client FGD–Southern Zone).

“*Most people do not believe the results and some do not believe a disease like HIV can affect them*. *So*, *if they get tested and they are told the results is positive they have to go for retesting*. *Some people can retest as much as four time and at different place and still do not believe the results*” (Client FGD–Northern Zone)

“*Those that find it difficult or those that don’t accept the results will always end up at different facilities where they are not known*” (Health worker FGD–Northern Zone).

Other reasons were: as part of health care processes (114 respondents). This was supported by the health worker focus group discussions held.

“*Some of the retesting is from our part. They give them the referral without telling them why they are being referred to Korle-Bu. When they come, they hand over their referral letter and when you ask them why they ae referred here, they will tell you I was asked to come here. When you open the letter, you will realize this person is positive client. Retesting comes in for you to be sure of the referral letter. In which I know is not the fault of the client*” (Health worker FGD–Southern Zone)

Check viral load (66 respondents). A few retested because they expected they would be cured after taking the medicine (15). Respondents explained that some clients assume the virus leaves their system after taking the medication for a long time so the test to see if the virus is gone.

“*To check if the virus is still there because I had taken the medicine for a long time”* (Client FGD- Middle Zones).

*“Some clients are retesting because they have been on the ARV for a long time and they have no medical issues*, *they believe because the virus is no more or not detected*, *they decide that its negative*” (Health worker FGD–Southern Zone).

While some also retested because their pastors had advised them to or felt they had been healed through a miracle (15 respondents). During the focus group discussions, respondents shared their personal experiences on this.

“*I have encountered several people who said the pastor said they are healed so they came for retesting*” (Health worker FGD—Southern Zone).

“*Some pastors will advise that you ignore the results and adhere to prayer and fasting*. *I have been a victim here*. *I fasted for 40 days and still tested positive so I’ll encourage anybody who is positive to be on medication*. *If you stick to medications*, *you can do any work that you want to do for as long as you want*” (Clients FGD–Middle Zone).

## Discussion

This study aimed to determine the prevalence of retesting and understand the main reasons for retesting among PLHIV in Ghana with the goal of improving monitoring, evaluation, and national programming.

Overall, 32.9% of the respondents at ART clinics and 53.1% of the clients profiled at HTS sites reported that they had ever retested for HIV after their initial diagnosis. The data shows that more than a third of the interviewees at ART clinics and over half of those profiled at HTS sites retested for HIV. The prevalence at HTS sites was similar to studies conducted in Malawi [9] and South Africa [8]. However, it was higher than previous studies in Tanzania (2.0%) and Ethiopia (13.2%) [4, 7]. In this study, the participants who visited HTS centers on average took two additional HIV tests after their initial positive diagnosis.

Between 2014 and 2019, the annual prevalence of retesting among all surveyed individuals at ART centers ranged from 8.1% to 11.3%. However, those diagnosed within these same years, the prevalence of retesting was higher, ranging from 22.3% in 2017 to 25.0% in 2015. This finding supports the high rate of retesting after initial diagnosis [4, 7, 10–12]. Participants in focus group discussion confirmed this, stating that many newly diagnosed clients who are either in denial of their status or doubt the test results opt for retesting to confirm their diagnosis. Among clients who retested in 2019, approximately half informed the service provider that they had already been diagnosed and about 90% used the same name during testing. The statistics for other years (2014 to 2018) were also similar. This study provided a unique opportunity to accurately capture retests at both ART clinics and HTS sites and avoiding double counting of individuals who have already been tested. During health worker focus group discussions, health workers mentioned that even when clients informed them that they are retesting or came with referral letters, they still recorded them as new entries because the current data entry forms do not allow for retesting. It is also important to increase client awareness about the implications of retesting so they feel more comfortable disclosing their retesting to service providers.

Furthermore, the highest prevalence of retesting from 2017 to 2019 was observed among individuals aged 18–30 years. This finding is not surprising given that clients within this age group are young and in the prime of their lives. While it is recognized that young people engage in riskier sexual behaviors that increase their chances of contracting HIV, they are also disproportionately affected by the emotional impact of an HIV diagnosis. It is common for them to doubt their diagnosis and take longer to accept it due to the stressors associated with being diagnosed with HIV at a young age [15–17]. This finding presents an opportunity for targeted interventions aimed at reducing retesting rates among youth and ensuring that individuals seeking to retest indicate their status, thereby ensuring that their data is captured appropriately. Previous studies have indicated that youths diagnosed with HIV are more likely to be lost to follow-up during treatment [17]. Without targeted interventions, both youth and individuals who repeatedly seek testing may neglect their treatment.

Study participants provided various reasons for retesting including the need to confirm their diagnosis, spiritual reasons (such as feeling cured after spiritual encounters), and routine health care practices. These reasons align with those reported in previous studies conducted in multiple settings [4, 6, 7, 10–12].

## Limitations

The major limitation of the study is that the results are based on clients’ self-reported HIV testing. This means the results could be affected by recall bias due to the longer recall period when they retested, the number of times a client retested, or even whether they retested at all. We had no way of verifying their reports. However, by focusing some of the analysis solely on the year 2019 (the year in which the study took place), we were able to mitigate some of the biases associated with failure to recall information accurately. The fact that our study covered the entire nation with a relatively large sample size gives us confidence in the reliability of our estimates and suggests that they can be extrapolated to all parts of the country.

## Conclusion

Overall, the prevalence of retesting among study participants already receiving antiretroviral therapy was found to be high (32.9%) as was the prevalence among those accessing HIV testing services (53.1%) over the past six years. Reasons such as doubt, denial, and faith, often lead individuals to undergo repeated testing, sometimes as many as 11 times. However, all of these retesters are counted as new clients, which inflates the HIV positivity rate and results in an overestimation in the country as other studies have also noted [18]. While it may be difficult to prevent clients from retesting, the implementation of well-planned interventions to accurately account for the high number of retesting could have a positive impact, bringing us closer to more accurate figures and improving the country’s linkage to care rate. These efforts can contribute to Ghana’s progress towards achieving the 95-95-95 targets.

## Recommendations

Based on these findings, we recommend that the National AIDS/STI Control Program implement interventions to increase knowledge about the importance of informing service providers about retesting. These interventions should be targeted at HTS sites and among the youth. Additionally, all newly diagnosed clients should be profiled at HIV testing sites. It would also be beneficial for the Ghana Health Service (GHS) to expedite the digitization and networking of sites, which is currently underway. In the meantime, it is crucial for the NACP to revise the data capturing tool (both HTS and linkage registers) to accurately record retesting instances at all levels of HIV healthcare provision. Providers should be educated about the potential for delayed linkage to care due to repeat testing and should counsel clients accordingly. Furthermore, providers should be encouraged to maintain a positive attitude towards clients who disclose retesting.

## Supporting information

S1 File (XLS)
